# Herbal Medicines Useful to Treat Inflammatory and Ulcerative Gastrointestinal Disorders: Preclinical and Clinical Studies

**DOI:** 10.1155/2018/3605748

**Published:** 2018-03-29

**Authors:** Sérgio Faloni de Andrade, Luisa Mota da Silva, Shahram Golbabapour, Steve Harakeh

**Affiliations:** ^1^Núcleo de Investigações Químico-Farmacêuticas (NIQFAR), Universidade do Vale do Itajaí (UNIVALI), Rua Uruguai, No. 458, Centro, 88302-202 Itajaí, SC, Brazil; ^2^Shiraz Transplant Research Centre, Shiraz University of Medical Sciences, Shiraz, Iran; ^3^Special Infectious Agents Unit, King Fahd Medical Research Center, King Abdulaziz University, Jeddah, Saudi Arabia

Gastrointestinal disorders are among the most common illnesses that affect people nowadays. Their prevalence and incidence have been on the rise during the last decade. This high prevalence and incidence are due to the contemporary lifestyle we live. Such lifestyles include bad dietary habits, consumption of drugs, alcoholic drinks, and stress. It is very common that gastrointestinal disorders are characterized with inflammatory and ulcerative processes from the stomach or gut.

The main inflammatory and ulcerative disorders associated with the gastrointestinal tract include gastritis, ulcers, colitis, Crohn's disease, and mucositis. These disorders are difficult to treat. The recurrence and side effects are very common after treatment with available drugs. Based on that, there is an urgent need for the search for more effective and safe pharmacological options for the treatment of inflammatory and ulcerative gastrointestinal disorders. In the recent years, plant extracts and natural products have been sought for the important role in the treatment of the previously mentioned disorders. Thus, a great deal of effort and research has been undertaken to find suitable natural plants and compounds with proven potential. For this reason, hundreds of plant extracts and isolated active compounds have shown a promising potential. However, for the majority of them, it is necessary to conduct preclinical studies followed by the clinical studies in humans in order to be approved for human use. This special issue is focused on this topic. The edition consists of seven articles including a clinical study, three preclinical studies, and three reviews, which are briefly described below.

The clinical study was performed by B. Liu et al., who reported that the use of Chinese medicinal herbs mix composed of seven plants (CIF) and mesalazine in the treatment of ulcerative colitis (UC). In this study, 60 patients with chronic UC were treated only with either oral mesalazine or mesalazine in combination with CIF enema. The results showed that combination of mesalazine and CIF significantly improved the clinical symptoms, the colon mucosal conditions, the Mayo Clinic Disease Activity Index, and quality of life, when compared to mesalazine alone.

Considering the preclinical studies, one of the contributions is the work of T. Mao et al., who explored the mechanism of Qingchang Wenzhong Decoction (QCWZD), preparation derived from eight plants, in UC in rats models. The authors showed that QCWZD administration significantly alleviated colitis-associated inflammation, upregulating serum macrophage-stimulating protein (MSP) and receptor d'origine nantais (RON) expression in the colon, reduced the pAkt (protein kinase B [Akt], phosphorylated [p] Akt) levels, promoted zona occluden 1 (ZO-1) expression, and depressed claudin-2 expression. Of this mode, it was possible to conclude that QCWZD appears to attenuate DSS-induced UC in rats by upregulating the MSP/RON signaling pathway, contributing to understanding of QCWZD benefits in UC.

Still considering the preclinical studies, Y. Yang et al. described the anti-inflammatory effects and the underlying mechanisms of GCZX-pill, Chinese herbal formula composed of six herbs, on trinitrobenzene sulfonic acid- (TNBS-) induced UC in rats. The results demonstrated that the GCZX-pill can attenuate colitis in rats and the anti-inflammatory effect of the GCZX-pill may be related to the reduction of enterochromaffin colonic cells hyperplasia and serotonin availability.

The third preclinical study was done by H. Zhang et al., who tested the effects of two Chinese herbal formulations, Erchen decoction (ECD) and Linguizhugan decoction (LGZGD), on insulin resistance in rats. The results indicated that ECD and LGZGD have protective effects against high-fat diet-induced liver insulin resistance and their underlying mechanisms involve the TNF-*α* and insulin pathway.

In the review articles, P. Ren et al. reported a comprehensive review concerning the efficacy and safety of Kangfuxinye enema (Chinese herbal medicine extracted from the* Periplaneta americana*) combined with mesalamine for the ulcerative colitis (UC) patients and in addition evaluated the grade of the quality of evidence by using the GRADE (grading of recommendations, assessment, development, and evaluation) approach. With this revision, the authors concluded that although Kangfuxinye enema seems to be effective and safe for treating UC patients in this systematic review, Kangfuxinye enema combined with mesalamine was weakly recommended due to very low to moderate quality of available evidence by the GRADE approach.

In another review, C. Wang et al. discussed another Chinese herbal formula known as Huangqin-Tang (HQT), composed of four ingredients: the roots of* Scutellaria baicalensis *Georgi,* Glycyrrhiza uralensis* Fisch,* Paeonia lactiflora* Pall, and the fruit of* Ziziphus jujuba* Mill. The authors concluded that available reports suggested that the efficacy of HQT or its components on UC is related to the intestinal environment improvement, immune modulation, and regulation of inflammatory pathways or cytokines. However, most of the data were based on animal studies or in vitro experiments; thus, the effects and mechanisms of HQT in UC patients remain to be explored or verified.

Finally, L. M. da Silva et al. did a review concerning the potential role of propolis in the treatment and prevention of gastrointestinal disorders. They reported and critiqued the studies showing the beneficial effects of propolis and its active compounds in the treatment of gastrointestinal diseases; however, they concluded that only few clinical trials have been conducted to prove their effectiveness and safety against human ulcers and their associated pathologies.

Thus, this special issue included different investigations related to the efficacy of herbal medicines in the treatment of inflammatory and ulcerative gastrointestinal disorders, based on clinical and preclinical studies as well as the revision of the literature related to the objective of this special issue.

*Sérgio Faloni de Andrade* *Luisa Mota da Silva* *Shahram Golbabapour* *Steve Harakeh*

